# The effects of intensive feeding on reproductive performance in laboratory zebrafish (*Danio rerio*)

**DOI:** 10.1371/journal.pone.0278302

**Published:** 2022-11-29

**Authors:** Isaac Adatto, Christian Lawrence, Lauren Krug, Leonard I. Zon

**Affiliations:** 1 Department of Stem Cell and Regenerative Biology, Harvard University, Cambridge, MA, United States of America; 2 Stem Cell Program and Division of Hematology/Oncology, Children’s Hospital Boston and Dana Farber Cancer Institute, Boston, MA, United States of America; 3 Harvard Medical School, Boston, MA, United States of America; 4 SmartLabs, Boston, MA, United States of America; Laboratoire de Biologie du Développement de Villefranche-sur-Mer, FRANCE

## Abstract

The zebrafish (*Danio rerio*) is among the most widely used model animals in scientific research. Historically, these fish have been reared in the laboratory using simple methods developed by home aquarists. For laboratories with high demand for breeding and generation turn-over, however, there has been a shift away from this approach towards one that leverages techniques, tools, and feeds from commercial aquaculture to help accelerate growth rates and decrease generation times. While these advances have improved efficiency, the effects of feeding zebrafish diets that are designed to grow disparately related cold-water fish species to market size quickly are not well-understood. To explore the impacts that intensive feeding protocols may have on this species, groups of zebrafish larvae from two different wild-type lines were stocked into treatment tanks at a standard density of 10 fish per liter and were administered either a “high” or “low” food diet for a maximum of 63 days. During their growth phase, the “high” food diet group received at least 8x more rotifers and at least 2x more *Artemia* than the “low” food diet group. Growth, survival, and reproductive performance (fecundity and viability) were measured in these fish and in their offspring. We found that fish that were fed more grew more rapidly and were able to reproduce earlier than fish that were fed less, but they were also more likely to produce higher proportions of non-viable embryos.

## Introduction

The growth in the usage of the zebrafish (*Danio rerio*) as a biomedical model organism has, to some extent, been underpinned by improvements and refinements in husbandry techniques and technology. When considering the reproductive potential of zebrafish, effectively and efficiently collecting thousands of viable embryos for research continues to be an important aspect of husbandry research for some laboratories. Early studies on successful breeding have focused on field observations [1, 2], male-to-female interactions, and complex behaviors by looking at the role of pheromones [3, 4], courtship behavior [5, 6], dominance [7], kin recognition [7–9], inbreeding depression [10–12] and spawning frequency [13, 14]. More recently, studies have looked at other factors that may impact reproductive performance as it relates to the domestication and housing. These have focused on effective stocking densities [15], stress and infection [16], tools for rapid embryo collection [17], diet and nutrition [18–22] and interruptions in circadian rhythm [23, 24]. Still, reproductive performance (fecundity and viability) may be dependent on a plethora of other indirect parameters, many of which have not yet been studied. Factors such as generational comparisons, light intensity, water conductivity, age and growth rates are all likely impacting performance in some way. Further complicating factors is the realization that in zebrafish facilities, these parameters all form an interconnected network that may influence entire colonies differently. Therefore, understanding the importance of these parameters in isolation is crucial to maximizing efficiency in any research operation.

The interactions between growth, nutrition and reproductive performance in zebrafish are not well understood and are the topic of vigorous debate within the zebrafish community. Field observations and detailed studies have helped inform our understanding of the nutritional requirements of zebrafish [1, 2, 25–27]. Consequently, diets used in zebrafish facilities have evolved over the past decades. Diets had traditionally included live feed, such as *Paramecium* and *Artemia* nauplii, in combination with dry flake food, for all life stages [27]. Over time, this approach has changed to include diets that target specific nutritional profiles and developmental stages such as Brachionid rotifers for larvae [28] and formulated commercial aquaculture diets for juvenile and adult stages. In addition, laboratory formulated diets are continually being developed and refined using cold-extrusion agglomeration, a technique that provides a product with a more stable, digestible and preserved nutritional profile [22, 29]. These changes have translated to a plasticity in growth and have led to shorter generation times. Recent studies have reported faster growth using various stocking densities, diets and feeding regiments that achieve a time to first reproduction of 57 days post fertilization (DPF) [28]. Generation times were shortened further to 47 DPF by Aoyama [30], and more recently reduced even further to 43 DPF [31, 32]. While there are obvious logistical benefits associated with shortened generation times, there is less focus on the potential deleterious effects of rapid growth on reproductive performance. In this study, we have attempted to directly manipulate growth rates to better understand if the rate of growth during early development is a potential factor impacting reproductive performance as a measure of viability and fecundity in age-matched zebrafish.

## Materials and methods

### Ethics statement

The institutional Animal Care and Use Committee at Harvard University approved all experiments in which animals were used (IACUC protocol # 11–22).

### Animals (F_0_ generation)

Fish from two commonly used zebrafish wild-type lines, AB and Tübingen, were set up in a spawning event. Fifty 4-month-old fish from each line were placed in separate breeding tanks at a 1:1 male to female ratio with a divider to prevent spawning from occurring overnight. The following day, the divider was removed at 10 AM and fish were allowed to spawn for 60 minutes. Fertilized embryos from each strain were collected, and transferred into petri dishes at a density of 35 fish per petri dish, and incubated at a room temperature of 25.5°C. On day 5, 420 larvae were randomly selected and arrayed into 12 3.5-liter treatment tanks at 35 fish per tank at a density of 10 fish per liter. Six tanks housed AB while the other six tanks housed Tübingen. At variable intervals, occurring more often during early stages of development, 10 fish were randomly selected and removed from each triplicate tank, and were placed in tanks measuring 9 cm x 16 cm with 150 mL of system water and photographed from above (Canon PowerShot *S*95 digital camera). The standard length of each of the sampled fish was determined by analyzing the photographs using ImageJ (version 2.0.0-rc-69/1.52i). At the same time, survival was measured by counting all fish in each tank.

### Dietary treatments, delivery (F_0_ generation)

**Fig 1:**
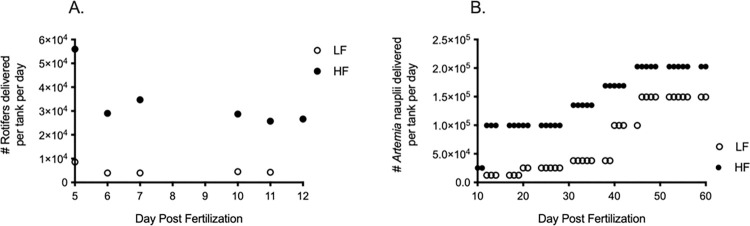
Rotifer & *Artemia* delivery per tank per day. (A) Daily rotifer concentration delivered per growth treatment, excluding weekends (day 8 & 9). Open circles = LF, closed squares = HF. (B) Daily *Artemia* nauplii delivered per growth treatment, excluding weekends. Open circles = LF, closed squares = HF.

Beginning at 5 DPF, AB and Tübingen fish were fed two concentrations of *Brachionus plicatilis*, an L-type saltwater rotifer harvested from continuous cultures and maintained on a rotifer diet of *Nannochloropsis* and *Tetraselmis* algae. Initial rotifer inoculations were fed out to all treatments at 5 DPF in 155 mL at a salinity of 0.5% to tanks off flow. Additional rotifers were fed out daily and suspended at a salinity of 0.5% water in 50 mL. Fish in the low food diet group (LF) were fed an initial inoculation of 8,500 rotifers followed by an average of 4,900 rotifers through 11 DPF. Fish in the high food diet group (HF) were fed an initial inoculation of 56,000 rotifers, approximately 6.5x more rotifers than the LF. From day 5 through day 12, fish in the HF were fed an average of 41,000 rotifers and at least 8x as many rotifers. While feeding on rotifers, all fish were kept in static tanks and not fed additional rotifers on corresponding weekend days, day 8 and 9 post fertilization. At 10 DPF, all tanks were placed on a recirculating system with a flow rate of 20 mL per minute (Fig 1).

Once tanks were put on the recirculating system, fish were fed two different concentrations of Great Salt Lake *Artemia* nauplii harvested from daily cultures. By hatching out an equal amount of *Artemia* cyst daily, consistent concentrations of *Artemia* were fed out to the two growth treatments via an automated feeding system (Tecniplast). Fish in the HF were fed *Artemia* nauplii beginning at 10 DPF, providing an overlap between rotifer and *Artemia* consumption. Fish in the LF were fed *Artemia* nauplii beginning at day 12 post fertilization. Initially, fish in the HF, received approximately 8x more *Artemia* than fish in the LF. Over the course of time fish fed *Artemia*, fish in the HF were fed 2x as many nauplii compared to fish in the LF. As fish aged, *Artemia* concentrations fed out to both growth treatments increased gradually, always feeding higher concentrations of *Artemia* to fish in the fast growth treatment until 60 DPF (Fig 1B).

Once fish were transferred to their new smaller housing tanks, delivery of all live diets was discontinued and replaced by two processed diets intended for adult fish. Fish were fed equally once daily an average of 70 mg of a 60/40 blend of Gemma Micro 500 (Skretting, France) and Otohime B2 (Japan), respectively.

### Time to first reproduction trial (F_0_ generation)

When >50% of the fish in triplicate tanks showed morphological sex differences [33], all fish from one tank in the triplicate were set up in a group cross. All fish in each tank were placed in a specialized tank [17] designed to promote zebrafish breeding behavior by reducing water depth, which has been shown to improve breeding in this species [17, 34]. Fish were set up in the evening, where all males were physically separated from all females by a barrier and were kept there for 20 hours until 10 AM the following morning. At this point, fish were allowed to spawn by removing the physical barrier and reducing the water depth to a shallow profile for 30 minutes. After the allotted time was completed, all fish were removed, and placed back into their housing tanks. If fish did not spawn, the second tank in the triplicate (tank B) for the given strain was set up in the breeding tank and spawned the following day under the same conditions. This sequence was repeated until fish successfully spawned any number of embryos. When fish spawned, embryos from each breeding tank were carefully harvested and placed in a 1 L tank filled with 500 mL of system water. After a 24-hour incubation at 25.5°C, the total number of live and dead embryos were manually counted and recorded at 7.5x magnification using a Leica M80 Stereo Microscope.

### Spawning trials (F_0_ generation)

At 60 DPF, five males and five females were randomly selected from tanks housing fish in the high food diet group and relocated into a smaller 1.1 L housing tank on recirculating flow (125 mL per min), thus keeping a 10 fish per L housing density equal to their rearing density. The same transfer was done to the low food diet group at 63 DPF. During this transfer, replicates and growth groups were kept separate ensuring no crossover of fish from one treatment group to another. Once fish were transferred into these new replicate tanks, they were allowed to acclimate to their new housing conditions for a minimum of 5 days before beginning spawning trials.

At week 11 post fertilization, all fish were set up once per week to spawn as described above. Fish were placed in spawning chambers by 2 PM, however genders were isolated with a separator inside the breeding tank, eliminating breeding overnight or at the onset of daylight the following morning. The following day, two and half hours after the onset of daylight in the facility, the separator was removed, allowing males and females to mix. Fish were placed in shallow water and were allowed to spawn for a 30-minute interval. After the allotted time was complete, fish were carefully removed and placed back into their original housing tanks. Before placing tanks back on flow, fish were weighed by transferring all animals into a 1 L tank with 200 mL of system water onto a tared balance (Ohaus, Model: Scout Pro SPE2001, Pine Brook, NJ). Netted fish were blotted dry prior to weighing. Post spawning weights were recorded and fish were then transferred back to their housing tanks. Embryos from each spawning chamber were collected and placed in a 1 L tank filled with 500 mL of system water and incubated at 25.5°C. Twenty-four hours after each group spawned, the total number of live and dead embryos were manually counted using a Leica M80 Stereo Microscope. Utilizing this methodology, each tank was set up weekly for five consecutive weeks, mimicking usage of laboratory zebrafish where spawning can be demanding. This was followed by a five-week rest period before resuming spawning for an additional three weeks.

### Transgenerational effects

To determine if the effects of varying feeding intensity during development would transmit to the next generation, every tank of fish used in the spawning trials was crossed to produce enough embryos to populate twelve (six per line) new experimental tanks of 35 fish at 10 fish per liter.

Fish were raised to 90 DPF, when morphological differences between the sexes were apparent in >50% of the fish. At this point, five males and five females were randomly selected from each tank and relocated into a smaller 1.1 L housing tank on recirculating flow (125 mL per min), thus keeping housing density equal to their rearing density (10 fish per liter). Transferred fish were allowed to acclimate to their new housing condition for two weeks before beginning F_1_ generation spawning trials.

### Dietary treatments (F_1_ generation)

At 5 DPF, the same type-L saltwater rotifer (*Brachionus plicatilis*) was delivered to all larvae, except concentrations did not vary and all tanks were fed equal amounts. Initial inoculation in all tanks consisted of approximately 8,500 rotifers in 155 mL at a salinity of 0.5% and 300 μL of rotifer diet. From day 6 through day 11 post fertilization, an average of approximately 4200 rotifers were delivered. At 10 DPF, all tanks were placed on a slow drip of a recirculating system (45 mL per min). At an average concentration of approximately 4200 per mL, first instar stage *Artemia* nauplii was equally delivered to all juvenile fish from day 10 post fertilization to day 90 post fertilization.

### Spawning trials (F_1_ generation)

At 104 days post fertilization, fish from both groups were set up and spawned in the same method as described above for the F_1_ generation, where fish placed in spawning chambers were set up with a divider isolating males from females. All fish were spawned in accordance with the exact same schedule as previously described, once per week for 5 weeks, 5 weeks rest, followed by once per week for 3 weeks. Twenty-four hours after each group spawned the total number of live and dead embryos were manually counted and recorded at 7.5x magnification using a Leica M80 stereo microscope.

### Statement of ethical approval

All fish used in the trials above were housed in a 10,000 L recirculating system with a constant conductivity of 1250 μS/cm, pH 7.5 and a water temperature of 28.5°C. At the end of the experiment, all animals were euthanized by rapid thermal shock in 2–4°C system water for a minimum of 30 minutes to ensure cessation of all opercular movement [35]. The use of all fish in the current study was performed in accordance and approved by the Institutional Animal Care and Use Committee at Harvard University (IACUC protocol no. 11–21).

### Statistical analysis

Since each spawning trial was a separate event from a previous week(s), spawning trials were treated as separate events. To accurately compare differences in spawning performance within each week, a two-tailed T-Test was used to calculate significant differences (*p* < 0.05) using GraphPad Prism v.5. Spawning performance included fecundity (total live embryos spawned) and viability (percent of viable embryos 24-hours after spawning).

## Results and discussion

### Zebrafish growth and survival–F_0_ generation

**Fig 2:**
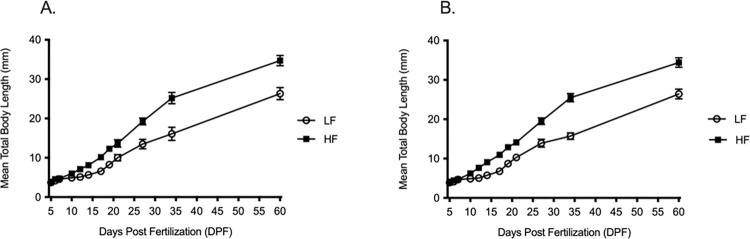
Growth curves of two F_0_ wild-type strains from 5 to 60 DPF. Mean total body length for two F_0_ generation wild-type strains, (A) AB and (B) Tü. Growth curves are represented by mean ± standard deviation. Open circles = LF, closed squares = HF.

All experimental fish used showed high survival rates with only minor corrections in rearing densities occurring at 5 DPF prior to transferring fish from petri-dish to 3.5-liter tanks. AB wild-type and Tübingen (Tü) zebrafish from each group grew at nearly identical growth rates. Within the first 35 DPF, AB and Tü strains from the LF grew 0.44 mm day^-1^ and 0.43 mm day^-1^, while the HF group grew at 0.74 mm day^-1^ and 0.75 mm day^-1^, respectively. When extending growth period to 60 DPF, AB and Tü strains from the HF grew at a rate of 0.60 mm day^-1^, while the LF grew at a rate of 0.43 mm day^-1^. At 60 DPF, fish length from the two groups were significantly different. Mean length for AB fish measured 34.7 mm ± SD 1.3 mm for the HF and 26.3 mm ± SD 1.6 mm for the LF. Similarly, mean length for Tü fish in the HF measured 34.4 mm ± SD 1.2 mm and 26.4 mm ± SD 1.2 mm for the LF (Fig 2).

### Spawning trials–F_0_ generation

While sexual dimorphism was already apparent in most fish from the HF at 40 DPF, the first clutch of embryos was not collected until 45 DPF, after the second attempt at spawning. The first embryos collected were from AB HF fish and measured a mean length of 31.4 mm ± SD 0.78 mm and mean weight of 0.26 g. A total of 14 embryos were collected with only 7% viability. Embryos from the Tü–HF were collected at 53 DPF, the fifth attempt at spawning, with a mean length measuring of 34.2 mm ± SD 1.07 mm and mean weight of 0.30 g. Here, 171 embryos were collected with 57% viability. Most fish in the LF did not begin to show sexual dimorphism until about 60 DPF. However, neither group spawned until 63 DPF, which was their 8^th^ attempt at producing embryos. AB wild-type fish from the LF with a mean length of 28.3 ± SD 0.82 spawned 1092 embryos, with 53% viability. Tü fish from the LF had a mean length of 28.0 ± SD 0.85 spawned 29 embryos with 62% viability (Table 1).

**Table 1:** Initial reproductive performance of fish from LF and HF. Values recorded at first successful group spawn of 35 fish. Fish were allowed to spawn for a total of 30 minutes.

Group	DPF at 1st spawn	Attempts to achieve embryo production	Mean body length (mm±SD)	Mean weight per fish (g)	Total # of embryos spawned	Viability (%)	Fecundity (# of live embryos spawned)
AB—HF	45	2nd attempt	31.4 ± 0.78	0.26	14	7	1
AB—LF	63	5th attempt	28.3 ± 0.82	0.21	171	53	576
Tü—HF	53	8th attempt	34.2 ± 1.07	0.30	1092	57	97
Tü—LF	63	8th attempt	28.0 ± 0.85	0.21	29	62	18

**Fig 3:**
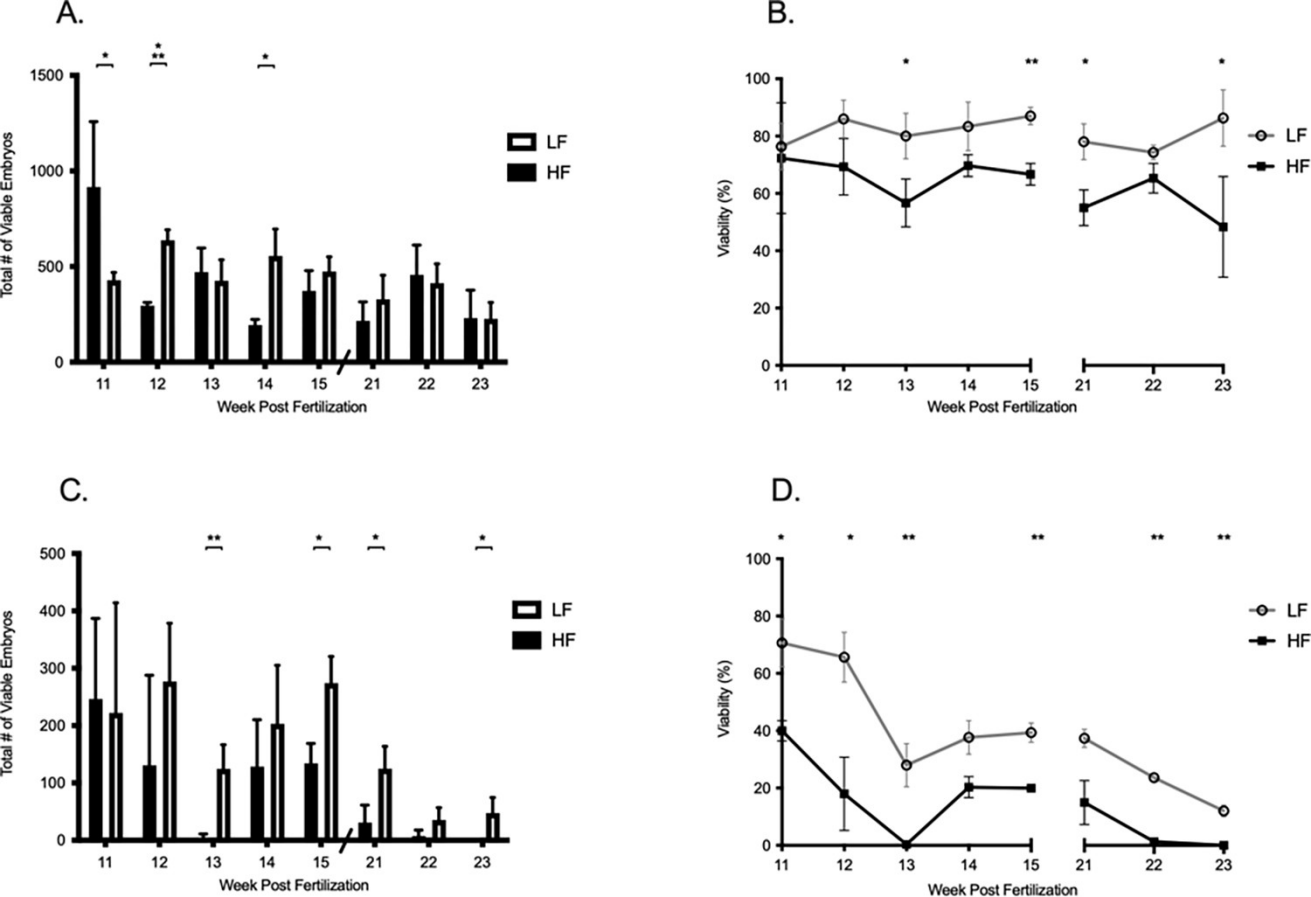
Measurement of reproductive performance in AB wild-type fish, F_0_ generation. Reproductive performance for the F_0_ (A, B) AB wild-type strain and (C, D) Tü wild-type line. (A) Fecundity is defined as the total number of live embryos spawned and (B) viability is defined as the percentage of live embryos at 24-hours post fertilization. Data shown as the mean ± standard deviation. Significant differences at each week are noted with * indicating *p* ≤ 0.05, ** indicating *p* ≤ 0.01, and *** indicating *p* ≤ 0.001 by successive two-tailed T-Test, n = 3 represented by 30 fish from each growth treatment.

At 11 weeks post fertilization, AB–HF spawned 2749 embryos, more than double the number of embryos spawned by AB–LF, 1286 embryos. Although, mean viability in this initial spawn was more similar, 72% vs. 76% respectively. Fecundity measured from the first embryos collected was the only week the measure of total live embryos spawned in AB–HF was significantly greater than the AB–LF (*p* = 0.070). Mean fecundity in four out of the eight spawning trials was greater in AB–LF and was significantly different in weeks 12 and week 14 post fertilization (*p* = 0.001, 0.011 respectively). In total, the LF spawned 997 more embryos during the eight-week spawning trials than the AB–HF. Viability measured in the LF was greater for all eight weeks spawned and showed a significant difference in four out of the eight weeks spawned (week 13, *p* = 0.025; week 15, *p* = 0.002; week 21, *p* = 0.012; and week 23, *p* = 0.031) (Fig 3A, 3B). Although the AB–HF fish weighed more throughout the spawning period, the differences in fish weight were not significant from week to week (S1 Fig).

Unlike AB fish, total embryos spawned by Tü at day 53 post fertilization were more similar regardless of how they were fed (Tü–HF = 739 embryos, Tü–LF = 666 embryos). However, viability in the HF was poor; 40% vs. 71% respectively. Mean fecundity in seven out of eight spawning trials were greater in Tü–LF compared to Tü–HF. Significant differences were measured in weeks 13 (*p* = 0.008), week 15 (*p* = 0.014), week 21 (*p* = 0.030) and week 23 (*p* = 0.038). In total, Tü–LF spawned 1879 more embryos during the eight-week spawning trials than the Tü–HF. The viability measured in the Tü–LF was also greater than the Tü–HF group in all eight weeks. In addition, significant differences were measured in six out of eight weeks (week 11, *p* = 0.028; week 12, *p* = 0.037; week 13, *p* = 0.0 21; week 15, *p* = 0.005; week 22, *p* < 0.0001; week 23, *p* = 0.005 (Fig 3C and 3D). Like the AB–HF group, the Tü–HF fish weighed more throughout the spawning period, but there were no significant differences in fish weight from week to week (S1 Fig).

After completion of our spawning trials, fish in the F_0_ generation were kept on the same feed regimen, receiving feed once per day. At 7 months post fertilization approximately 10 out of the 15 F_0_ females from the HF in both strains exhibited distended abdomens suggesting possible egg-associated inflammation [36]. In fact, females in the HF weighed 30% more in AB and 45% more in Tü than females in the LF from either group. Attempts to pair-wise spawn F_0_ fish resulted in higher spawning success, greater fecundity, and improved viability in the LF. These results suggest that fish reared with slower growth rates can still outperform fish that grow faster during development, especially beyond early maturation stages.

### Zebrafish growth and survival–F_1_ generation

**Fig 4:**
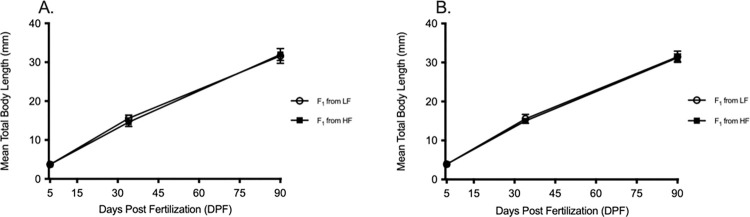
Growth Curves of two F_1_ wild-type lines from 5 to 90 DPF. Growth curves for two F_1_ generation wildtype lines (offspring of F_0_), (A) AB and (B) Tü from 5 to 90 DPF. Slow growth curves are represented by mean ± standard deviation. Open circles = LF from F_0_ LF parents, closed squares = LF from F_0_ HF parents.

Like the F_0_ generation, all experimental fish used in the F_1_ generation showed high survival rates with only minor corrections in rearing densities occurring at 5 DPF prior to transferring fish from petri-dish to 3.5-liter tanks. Similar growth rates were measured for all F_1_ generation AB wild-type and Tü zebrafish. Within the first 35 DPF, all AB wild-type grew 0.41 mm day^-1^, representing 0.3mm slower than AB–LF in the F_0_ generation, while the Tü strain grew 0.40 mm day^-1^, 0.2mm slower than the F_0_ generation. At 60 DPF, all fish in the F_1_ generation were smaller than the F_0_ generation at the same time point, and maturation was far from complete as sexual dimorphism was not obvious. Total growth calculations were extended 30 days later to 90 DPF and measured to be a total growth rate of 0.32 mm day^-1^ in both strains. At 90 DPF, mean fish length in all four groups was nearly identical (AB—31.97 mm ± SD 1.6, 31.63 mm ± SD 1.9, Tu—31.23 mm ± SD 0.98, 31.48 mm ± SD 1.45 mm (Fig 4).

### Spawning trials–F_1_ generation

**Fig 5:**
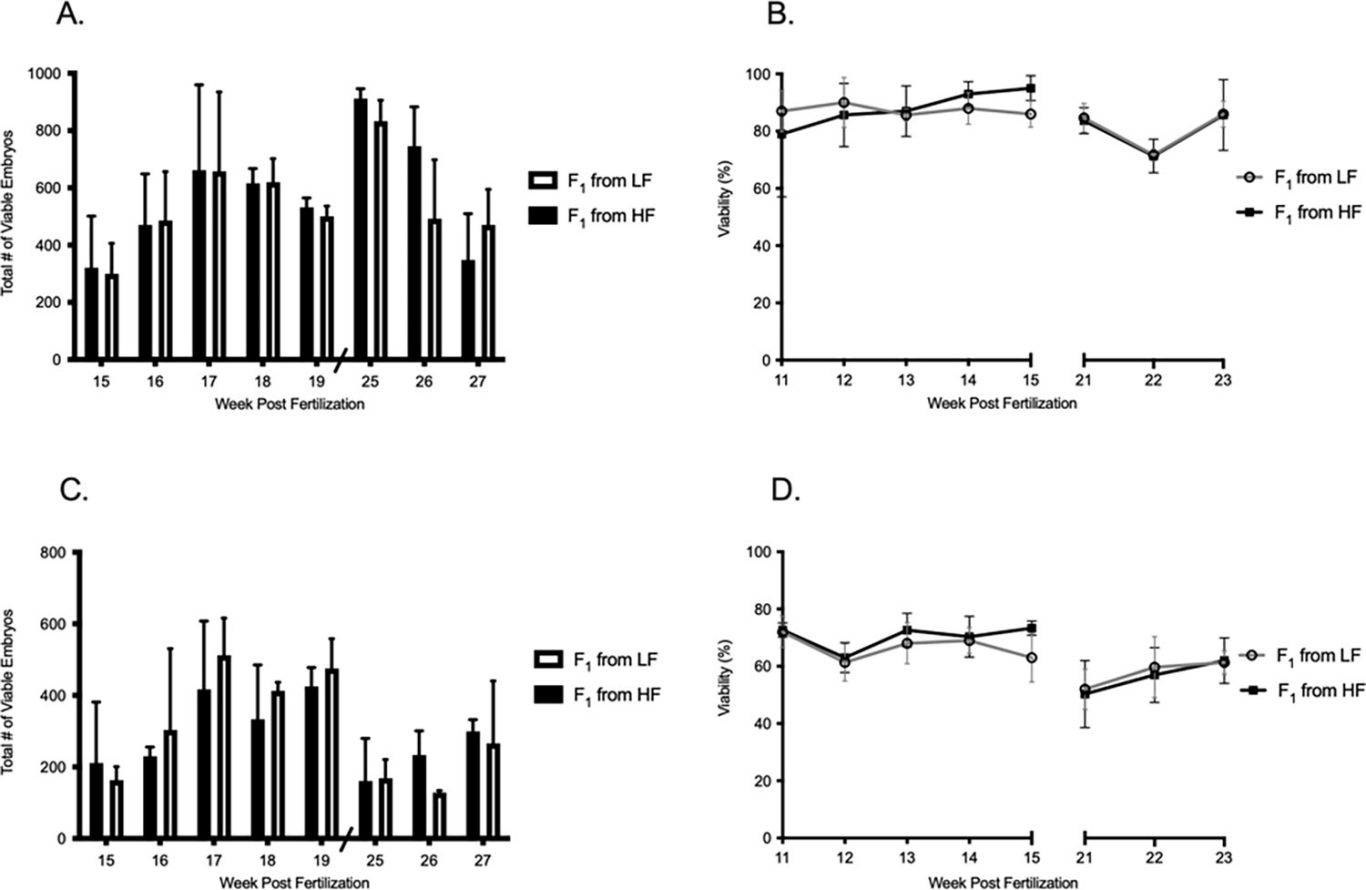
Measurement of reproductive performance in Tü wild-type fish, F_1_ generation. Reproductive performance for the F_1_ (A, B) AB wild-type strain and (C, D) Tü wild-type strain. (A) Fecundity defined as the total number of live embryos spawned and (B) viability defined as the percentage of live embryos at 24-hours post fertilization. Data shown as the mean ± standard deviation. No significant differences were measured in either fecundity or viability, n = 3 represented by 30 fish from each growth treatment.

The F_1_ generation showed no differences in reproductive performance when compared with the F_0_ generation. The total number of live embryos spawned for the F_1_ generation from AB–HF parents was 13820 embryos, whereas the F_1_ generation from AB–LF parents produced 13076 embryos. Viability was also found to be consistent from week to week, with a total mean viability for the eight weeks of 87% ± SD 7% in the F_0_ from AB–HF and 85% ± SD 5% F_1_ from AB–LF (Fig 5A, 5B). Because the two treatments in the F_1_ generation were maintained on the same diet, fish weight in the AB and Tü groups remained similar (S1 Fig).

The reproductive performance in Tü zebrafish showed similar patterns. Total fecundity for the F_1_ generation from Tü–HF parents was 6931 embryos, whereas the F_1_ generation from Tü–LF parents produced 7292 embryos. Week to week viability was also uniformly matched and no significant differences were measured. Total mean viability for the eight weeks measured 65% ± SD 9% in the F_1_ from Tü–HF and 63% ± SD 6% F_1_ from Tü–LF (Fig 5C, 5D).

### Viability comparison of F_0_ and F_1_ generations

**Fig 6:**
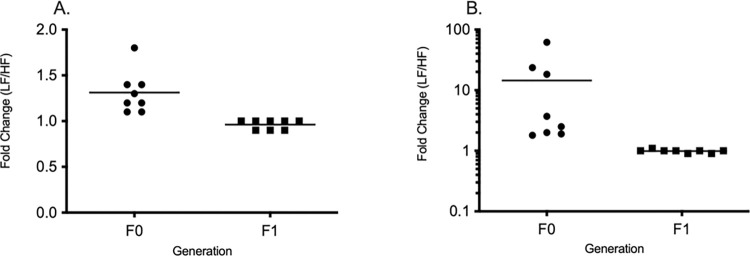
Fold change of embryo viability. Embryo viability measured from the F_0_ and F_1_ generations were used to calculate the fold change of the LF/HF in both wild-type strains (A) AB, (B) Tü. Values that deviate from 1.0 demonstrate a greater difference in reproductive performance.

To gain an understanding of the difference in viability from the F_0_ generation compared to the F_1_ generation, a fold change comparison was created by plotting the mean viability of the LF divided by the HF per strain. Plotted values calculated from the F_0_ generation show high variability and deviate largely from 1. This indicates noteworthy differences in reproductive performance caused by difference in growth rates. Whereas plotted values calculated from the F_1_ generation show little to no change from 1 (Fig 6).

Using live feeds for the first 63 days post fertilization, this study aimed to identify differences in reproductive performance induced by different feed rates in zebrafish. Feeding practices, enhanced diets and improved management have all led the way to help shorten generation times from what has been initially reported [37]. Here, we demonstrate how growth rates can be controlled without altering rearing density, sacrificing water quality, or eliminating a natural circadian rhythm [31, 32]. Faster time to first reproduction and maturation to adulthood are just a few of the benefits of rearing zebrafish with a high food diet. However, rapid growth also comes at an unwanted cost to a zebrafish research operation in the form of declining reproduction and an overall shortened window for embryo production. On the other hand, slower growth rates capable of growing mature fish in 63 DPF, while act to delay time to maturation, results in improved reproductive performance in zebrafish.

At the start of the spawning trials, fish from the HF were different in size (weight and length) than fish from the LF. Fish from the HF weighed 20% or more than the LF and grew 30% more in length. While fish in the HF were first to spawn and even outperform in fecundity (see F_0_ AB–Fast, week 11), we also observed a declining trend in reproductive performance for AB fish in the HF and an even steeper decline from Tü fish in LF. More importantly, viability in the LF statistically outperformed fish from the HF in 50% or more of the spawning trials. Fecundity was also statistically greater in 38% or more of the spawning trials in the LF (Fig 3).

It is well established that breeding performance of zebrafish is highly influenced by nutrition [38, 39]. The correlation of the two can be observed in recent studies where authors report growth rates along with reproductive performance. Forbes [19] reported smaller-sized diameters in embryos for those zebrafish fed to satiation up to 28 DPF, and reported a significant correlation between yolk mass diameter and hatch rate. Smaller sized embryos were subsequently followed with inferior quality and lower hatch rates than embryos spawned from fish fed less. In Castranova *et al*. [15], eight zebrafish research laboratories participated in a collective study that showed that the lab able to detect sexual dimorphism the quickest was also followed by the lowest % viable embryos, regardless of stocking densities. In another study by Lawrence *et al*. [40] fish with the lowest mean total length and weight values at the end of the feeding trials showed the highest viability rates when spawned. In Monteiro *et al*. [22] study, fish that were fed a diet resulting in the highest rates of growth produced clutches of embryos with lower viability than did the groups of fish grew more slowly. Newman et al. [41] reported on the influence of dietary intake of underfed-malnourished fish and found higher breeding success on those more well-nourished fish. Although the focus of these studies are not identical to the present study, these findings corroborate our observations; that diets and/or feed regimens have an impact of breeding performance and those that promote accelerated growth do not often support improved reproductive performance.

One potential causative factor could be related to the proportion of essential fatty acids (EFA) available within the diet consumed, or lack thereof [42, 43]. EFA requirements of fish differ from species to species and are dependent on several factors [41]. Several studies have showed an association with EFA deficiencies and spawning rate. EFA regulate the release of prostaglandins, which have an important role as pheromones, and also affect ovulation in fish. Therefore, zebrafish female with dietary EFA deficiencies may have a disrupted prostaglandin signaling pathway, ultimately preventing the release of eggs that can accumulate and degrade over time [27, 43].

Accelerating the time to maturation by overfeeding seems to also accelerate the development of gonads [44]. Previous studies have reported that puberty in female zebrafish is more dependent on fish size rather than age, where rapid gonad development is correlated with rapid growth rates [13, 45]. Yilmaz *et al*. [46] was the first to demonstrate proteomic profiles of egg quality in zebrafish. Using liquid chromatography and mass spectrometry, they determined that poor quality eggs were deficient in various proteins, including vitellogenin products, preventing eggs from going through complete maturation. In addition, mitotic germ cells in bony-fish (teleost) do not contain a limited number of developing eggs but rather continually produce them over their lifetimes [47]. Eventually, oocyte degeneration, or atresia, plays a major role in the number of healthy eggs produced [48, 49]. In our study, dissection of large egg-bound female zebrafish do not reveal a sac full of eggs, but rather fatty tissue, ovarian fluid and necrotic egg tissue, which often develops into a plug clogging the oviduct [50, 51]. This result may be explained by a combination of complex interactions between lipids, vitellogenesis and atresia. Vitellogenin is a yolk precursor primarily produced in the liver, transported by the blood, and continually taken up by developing oocytes [52, 53]. Since zebrafish are classified as asynchronous spawners, oocyte maturation and ovulation can be found at different stages of development [44, 48]. While vitellogenin has been detected in all tissues except the intestines, when detected in increasing levels, it does not accumulate in the liver but rather in the bloodstream and in the peritoneal cavity of zebrafish [54, 55]. Lipid consumption also plays a role in egg production and viability. Because zebrafish are lower-trophic-level consumers, they also have a lower dietary requirement, which may affect the way in which lipids are utilized for energy. Underutilized lipids may end up as ectopic fat storage, which may negatively impact spawning performance. While fish do store some fat in the visceral, subcutaneous, and intramuscular regions [27], fat storage is nominal compared to somatic and ovarian growth [44]. The latter is prioritized once the fish has met certain physical conditions in somatic growth and has attained sexual maturity. In our study, post spawning fish weight was not statistically different from one feed group to another. Female fish in the HF group may have displayed targeted ovarian weight increase and oogenesis as demonstrated in previous studies [27, 44]. However, the weight increase fish in the LF group may have been due to a combination of both somatic and ovarian growth. While these factors accelerate oogenesis early on, they may also promote producing egg over-production and retention in females as they age. This is more commonly observed in intensively fed fish.

The importance of evaluating reproductive performance in the F_1_ generation was to elucidate if dietary treatment of parents would have transgenerational effect on its offspring. All fish in the F_1_ generation grew at the same rates as the parental generation on the low feed diet. There were no differences in viability or fecundity throughout the 8-week spawning trial (Fig 5). Fish fed a HF showed depressed spawning performance, but their offspring did not, at least when fed at a LF rate. This suggests that the effects are confined to the animals being exposed to those conditions and are not transmitted to the next generation (Fig 6). Mean viability and total live embryo count in both lines in the F_1_ generation show improved spawning performance compared to the F_0_ generation, providing even further evidence that feed rates affect timing of sexual maturation, gamete quality and length of reproductive success.

## Conclusion

Many studies evaluating spawning performance in zebrafish state the importance of diet and nutrition [27, 38]. This is the first study to focus on equal diet and nutrition but fed at different quantities to produce two distinct growth curves. Even a well-balanced diet fed incorrectly can have profound negative effects. From these results we now have an enhanced perspective on the importance of feed rates in zebrafish nurseries, which have a direct impact on growth, as well as viability, fecundity and ultimately the longevity and health of the cohort.

As the zebrafish community continues to acquire more data on management leading to healthier, better performing fish, there is still more to learn from the more refined management methods established for other fish species grown in the aquaculture industry. A relevant example is seen in the management practices of broodstock vs. market fish of the same species [56]. Rapid growth past a certain size using optimized food conversion ratios is of importance for market fish. On the other hand, broodstock fish undergo complex breeding programs and are managed in ways to maximize and control offspring production by regulating diet and nutrition, temperature and photoperiod [57]. Common to most zebrafish research facilities, fish are mostly managed equally (temperature, water quality, photoperiod, diet) regardless of their intended purpose. Perhaps the only differences in husbandry practices linked to feeding are evident throughout the various life stages (i.e., larvae, juvenile, adult). Our results suggest that a diet that promotes accelerated growth rates may not necessarily be optimal for reproductive performance. Despite the advancements made in zebrafish husbandry, the next boundaries to break through should not be focused on quicker time to maturation. Instead, it would be of greater benefit to define the diet and nutrition necessary for optimal development of gametes to better regulate zebrafish reproductive cycles and maximize performance over the long-term. In turn, this may help in defining more specific definitions on parameters to better influence spawning and for managing broodstock versus a maintenance stock in zebrafish.

## Supporting information

S1 Fig Post spawn total fish weight (mg). Total fish weight after each spawning trial is represented by • for fish in the HF group and □ for fish in the LF group. A = AB–F_0_, B = Tü–F_0_, C = AB–F_1_ and D = Tü–F_1_. No significant differences are reported in weight over the 8 weeks fish were spawned.
(TIF)

S1 File (XLSX)
