# Supplementary figures and images for: The effects of intensive feeding on reproductive performance in laboratory zebrafish (Danio rerio)
Source: PLoS One. 2022 Nov 29;17(11):e0278302. doi: 10.1371/journal.pone.0278302 (PMC9707780; doi:10.1371/journal.pone.0278302)

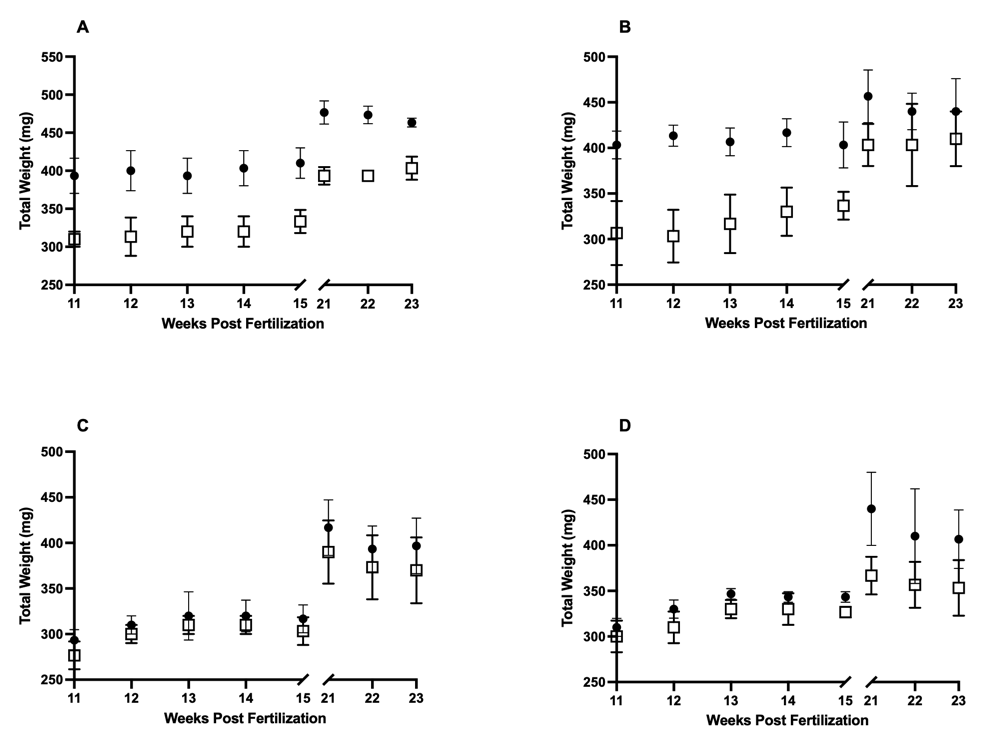

Supplement: S1 Fig — Total fish weight after each spawning trial is represented by • for fish in the HF group and □ for fish in the LF group. A = AB–F0, B = Tü–F0, C = AB–F1 and D = Tü–F1. No significant differences are reported in weight over the 8 weeks fish were spawned. (TIF) [file pone.0278302.s001.tif]
